# Predation-resistant *Pseudomonas* bacteria engage in symbiont-like behavior with the social amoeba *Dictyostelium discoideum*

**DOI:** 10.1038/s41396-023-01535-5

**Published:** 2023-10-26

**Authors:** Margaret I. Steele, Jessica M. Peiser, P. M. Shreenidhi, Joan E. Strassmann, David C. Queller

**Affiliations:** Biology Department, Washington University in St. Louis, St. Louis, MO USA

**Keywords:** Microbial ecology, Symbiosis, Soil microbiology

## Abstract

The soil amoeba *Dictyostelium discoideum* acts as both a predator and potential host for diverse bacteria. We tested fifteen *Pseudomonas* strains that were isolated from transiently infected wild *D. discoideum* for ability to escape predation and infect *D. discoideum* fruiting bodies. Three predation-resistant strains frequently caused extracellular infections of fruiting bodies but were not found within spores. Furthermore, infection by one of these species induces secondary infections and suppresses predation of otherwise edible bacteria. Another strain can persist inside of amoebae after being phagocytosed but is rarely taken up. We sequenced isolate genomes and discovered that predation-resistant isolates are not monophyletic. Many *Pseudomonas* isolates encode secretion systems and toxins known to improve resistance to phagocytosis in other species, as well as diverse secondary metabolite biosynthetic gene clusters that may contribute to predation resistance. However, the distribution of these genes alone cannot explain why some strains are edible and others are not. Each lineage may employ a unique mechanism for resistance.

## Introduction

Most eukaryotic organisms interact with bacterial symbionts throughout their lives. Some symbionts are vertically transmitted, such as the *Buchnera* endosymbionts of aphids [1], while others are acquired from the environment, such as the bioluminescent *Vibrio fischeri* symbionts of bobtail squid [2] or nitrogen fixing rhizobia of legumes [3]. These established symbioses are often facilitated by complex adaptations, such as specialized host structures for housing mutualistic symbionts. Similarly, parasitic symbionts often alter and exploit host structures to facilitate their own reproduction, as is seen in pathogens such as *Legionella pneumophila* and *Mycobacterium tuberculosis* that can replicate within phagocytic cells [4, 5]. However, these complex interactions likely originated from much simpler chance encounters or opportunistic infections. Predatory amoebae could be a source of novel intracellular pathogens because they constantly interact with bacteria in the environment and select for traits that contribute to survival of phagocytosis [6]. Following the same logic, amoebae may provide an opportunity to study the early stages of the evolution of mutualistic and parasitic bacterial symbionts.

The soil amoeba *Dictyostelium discoideum* is a simple, tractable organism that interacts with bacteria in different ways throughout its lifecycle. *D. discoideum* spends much of its life as a unicellular amoeba, consuming bacteria through phagocytosis. Although *D. discoideum* is capable of preying upon a diverse range of bacteria, predation-resistant bacteria have been identified in multiple phyla [7]. Aside from a few well studied human pathogens, little is known about the mechanisms these species use to escape predation. When *D. discoideum* amoebae run out of prey, tens to hundreds of thousands of amoebae aggregate to form a motile, multicellular slug. A fraction of these cells become sentinel cells, which eliminate potential bacterial pathogens by sequestering them within vacuoles and producing extracellular traps [8–10]. The slug migrates towards the soil surface, where it develops into a fruiting body that consists of a spore-filled sorus supported by a stalk, which aids in dispersal of spores by insects [11]. Dispersed spores hatch into amoebae when prey bacteria are present [12]. Bacteria capable of surviving phagocytosis, escaping the neutrophil-like activities of sentinel cells, and infecting the sorus may benefit from co-dispersing with spores or preying upon spores or hatched amoebae.

In addition to feeding on bacteria, *D. discoideum* acts as a host for both beneficial and pathogenic bacteria. *Paraburkholderia agricolaris*, *Paraburkholderia hayleyella*, and *Paraburkholderia bonniea* are conditionally beneficial intracellular symbionts of *D. discoideum* that may be acquired from the environment or vertically inherited. These bacteria survive intracellularly after phagocytosis and are therefore a poor food source for amoebae but they induce secondary infections by edible bacteria that otherwise would not infect the sorus [13, 14]. As a result, infection by *Paraburkholderia* allows *D. discoideum* spores to co-disperse with prey bacteria that can seed new populations, which is beneficial to spores that disperse to areas with limited prey [13, 15]. *D. discoideum* amoebae can also be infected by numerous human intracellular pathogens under laboratory conditions, making it a popular model system for studying bacterial pathogenesis [16–19]. In nature, amoebae sometimes act as environmental reservoirs for pathogenic bacteria such as *Bordetella bronchiseptica* [20] and *Mycobacterium bovis* [21]. However, far more is understood about the mechanisms human pathogens use to infect *D. discoideum* than is known about the bacteria it interacts with in nature.

A recent study of bacteria isolated from wild *D. discoideum* showed that many soil bacteria form short-lived associations with *D. discoideum* [7]. Many of these isolates belonged to genus *Pseudomonas*, including both edible and predation-resistant strains. To better understand the evolution of predation resistance and pathogen-like behaviors in soil *Pseudomonas* species, we tested fifteen *Pseudomonas* isolates for susceptibility to predation and ability to infect the sorus. These strains were isolated from wild *D. discoideum* and are therefore known to interact with the predator in nature. We used a combination of genome sequencing, microscopy, and infection assays to explore the evolution of predation resistance in soil *Pseudomonas* species and to look for parallels between the effects of predation-resistant *Pseudomonas* and symbiotic *Paraburkholderia*. This work revealed that some soil *Pseudomonas* species exhibit behaviors similar to symbiotic *Paraburkholderia* species, including the ability to infect *D. discoideum* throughout its life cycle and induce secondary infections.

## Methods

### Strains, media, and culture conditions

All bacterial strains, *D. discoideum* clones, and plasmids used in this study are listed in Supplementary Table S1. Thirteen *Pseudomonas* strains were isolated from *D. discoideum* fruiting bodies from amoebae collected from soil and deer feces at Mountain Lake Biological Station, Virginia in 2014 [7]. *Pseudomonas* strains Pf2 and Pf3 were isolated from wild *D. discoideum* strain QS161, collected from soil from Mountain Lake Biological Station, Virginia in 2000 [22]. Culture conditions are described in the Supplementary Methods.

### Quantification of bacterial CFU

Bacteria were collected from liquid or by flooding agar with 1 ml of KK2. Seven 1:10 serial dilutions were prepared and 10 µl droplets of each dilution were spotted in triplicate on LB agar. Plates were incubated at 30 °C and CFU were counted after 1-2 d.

### Edibility assays

To classify *Pseudomonas* strains as edible or predation-resistant, *D. discoideum* QS157 spores were spread on SM/5 agar plates with *Pseudomonas* sp. as a sole food source or with a 10:90 mixture of *Pseudomonas* sp. and food bacterium *Klebsiella pneumoniae*. As controls, QS157 was also grown on *K. pneumoniae*, *Pa. bonniea* Bb859 (a slightly edible symbiont species), and *Ps. aeruginosa* PAO1 (a predation-resistant pathogen). Plates were monitored for 7 d for fruiting body development. After 7 d, cells were collected to quantify the remaining bacterial CFU.

### Bacterial carriage assays and CFU per sorus

To determine whether *Pseudomonas* strains infect the sorus of *D. discoideum* fruiting bodies, *D. discoideum* QS157 spores were spread on SM/5 plates with either 100% *Pseudomonas*, 50% *Pseudomonas* and 50% *K. pneumoniae*, or 10% *Pseudomonas* and 90% *K. pneumoniae*. Individual sori were collected after 7 d and spotted onto the surface of SM/5 agar plates. The number of spots that showed bacterial growth was recorded to determine the fraction of sori infected by bacteria. To enumerate the bacteria per sorus, individual sori were transferred to PCR tubes containing 200 µl of KK2 supplemented with 0.05% NP-40 alternative. Tubes were vortexed to release spores and bacteria from sori, then bacterial CFU were quantified.

### Intracellular survival assay

To determine how long bacteria survived after phagocytosis, we used an intracellular survival assay similar to that described by Pukatzki et al. [23]. Axenically grown *D. discoideum* AX4 amoebae were washed three times with cold KK2. Washed amoebae were resuspended in HL5 at a concentration of 2 × 10^6^ cells/ml. 20P_3.2_Bac4, 6D_7.1_Bac1, 18P_8.2_Bac1, *Ps. aeruginosa* PAO1, *K. pneumoniae*, and *Pa. bonniea* Bb859 were grown overnight in LB broth at 30 °C, 225 rpm, then spun down and resuspended in HL5 at OD_600_ 1.

1 ml volumes of amoebae were transferred to wells of two 24-well tissue culture plates. After 1.5 h, 50 µl of bacteria were added to wells. The 24-well plates were centrifuged for 10 min at 750 rcf, then incubated at room temperature for 30 min. The supernatant was removed, leaving only attached cells, 1 ml of KK2 was added and then removed to wash the cells, then 1 ml of KK2 with 400 µg/ml gentamicin was added to each well to kill extracellular bacteria. Plates were left at room temperature for either 3 or 22 h to determine how long bacteria were able to survive within amoebae. The number of intracellular bacteria was determined by collecting the cells from each well and washing them three times with cold KK2 to remove the gentamicin. Cells were finally resuspended in 1 ml of KK2 with 0.05% Triton-X 100 to lyse the amoebae. Bacterial CFU were then quantified to determine the number of intracellular bacteria recovered from each well. The time between addition of gentamicin and lysis of amoebae was approximately 5 and 24 h for the two timepoints.

### Protection of other species

Three edible strains, 14P_8.1_Bac3-GFP, 7P_10.2_Bac1-GFP, and *K. pneumoniae*-GFP, and three predation-resistant strains, 20P_3.2_Bac4, 6D_7.1_Bac1, and 18P_8.2_Bac1, were grown overnight at 30 °C, 225 rpm in SM/5 broth, then diluted to OD_600_ = 1. Edible strains were mixed 1:1 with predation-resistant strains. Controls without predation-resistant bacteria were mixed with sterile broth. *D. discoideum* AX4 amoebae were grown axenically, then washed and resuspended in SM/5 broth at a concentration of 2 × 10^6^ cells/ml. 65 µl of bacteria and 15 µl of amoebae were spread on 60 mm SM/5 agar plates and incubated at room temperature. After 7 d, all cells on the plate were collected and bacterial CFU were quantified to determine the number of edible and predation-resistant bacteria remaining.

### Microscopy

The contents of sori infected with GFP-labeled *Pseudomonas* strains were examined using confocal microscopy to determine whether bacteria in the sorus are intracellular. *D. discoideum* QS157 was grown with *Pseudomonas* (10%) and *K. pneumoniae*, as described above. Sori were collected from fruiting bodies and suspended in 50 µl KK2 with 1% calcofluor. 10 µl was placed on top of a 1% agarose pad on a microscope slide, prepared by using a siliconized glass cover slip to flatten a 125 µl drop of agarose, then covered with a cover slip.

To visualize interactions between *Pseudomonas* and amoebae, microscope slides were embedded in petri plates under a thin layer of 0.5% agar SM/5. GFP-labeled *Pseudomonas* strains and *K. pneumoniae* were suspended in KK2 at OD 1.5. Each *Pseudomonas* strain was mixed with *K. pneumoniae* at a 1:1 ratio, then 200 µl of the mixture and 4 × 10^5^ QS9-mCherry spores were spread on agar over embedded slides. After approximately 42 h, slides were cut out of the agar plate, a cover slip was placed on top of the agar, and cells were imaged.

### Genome annotation

Thirteen *Pseudomonas* genomes were sequenced and assembled as described in the Supplementary Methods. The assembled genomes were annotated using the Rapid Annotation through Subsystem Technology (RAST) platform [24] and then re-annotated using the NCBI Prokaryotic Genome Annotation Pipeline [25]. AntiSMASH [26, 27] was used to identify clusters of genes encoding secondary metabolite biosynthesis pathways. These clusters were grouped using all-v-all BLAST to identify sequences that shared ≥70% nucleotide identity over ≥20% of the query length. Groups were visualized using Cytoscape v3.9.0 [28].

To determine whether the *Pseudomonas* genomes encode Type III secretion systems (T3SS), representative protein sequences for T3SS structural proteins SctJNQRSTUV, which are found in both T3SS and flagella; SctC, found only in T3SS; and FlgBC and FliE, found only in flagella [29], were downloaded from the NCBI protein database. BLAST+ v2.9.0 was used to search for homologous sequences within a local BLAST database containing proteins encoded by the *Pseudomonas* genomes. To identify Type VI secretion systems (T6SS), reference sequences for 13 structural proteins (TssA-TssM) were downloaded from the SecReT6 database [30]. CD-HIT v4.8.1 [31] was used to cluster sequences that shared more than 40% amino acid identity, and then reference sequences from each cluster were used to search for homologous sequences in the *Pseudomonas* genomes using protein BLAST. T3SS and T6SS in different genomes were grouped based on homology into three distinct T3SS and five T6SS. ExoU, ExoY, ExlA, and MgtC homologs were identified by using BLAST to search all proteins encoded by the *Pseudomonas* genomes for homology to reference sequences from *Ps. aeruginosa* (accessions ASM94169.1, PWU33926.1, QDL04633.1, and BAQ42388.1).

### Genome phylogeny and average nucleotide identity

Orthofinder v2.5.4 [32–34] was used to construct a species phylogeny based on 1734 orthologs found in 30 *Pseudomonas* genomes. Reference genomes were selected based on the best matches to the 16S rRNA genes. The phylogeny was visualized using the interactive Tree of Life (iTOL) v6 [35] and was further modified using Adobe Illustrator. To determine whether sequenced genomes belong to species with reference genomes in NCBI databases, Average Nucleotide Identity for each species pair was calculated using FastANI [36].

### Statistical tests

Fisher’s exact test was performed in R v4.2.0 [37]. Other statistical tests were performed using GraphPad Prism v9.5.1 (GraphPad Software, San Diego, California USA, www.graphpad.com).

## Results

### Predation-resistant *Pseudomonas* strains are not monophyletic

**Fig. 1:**
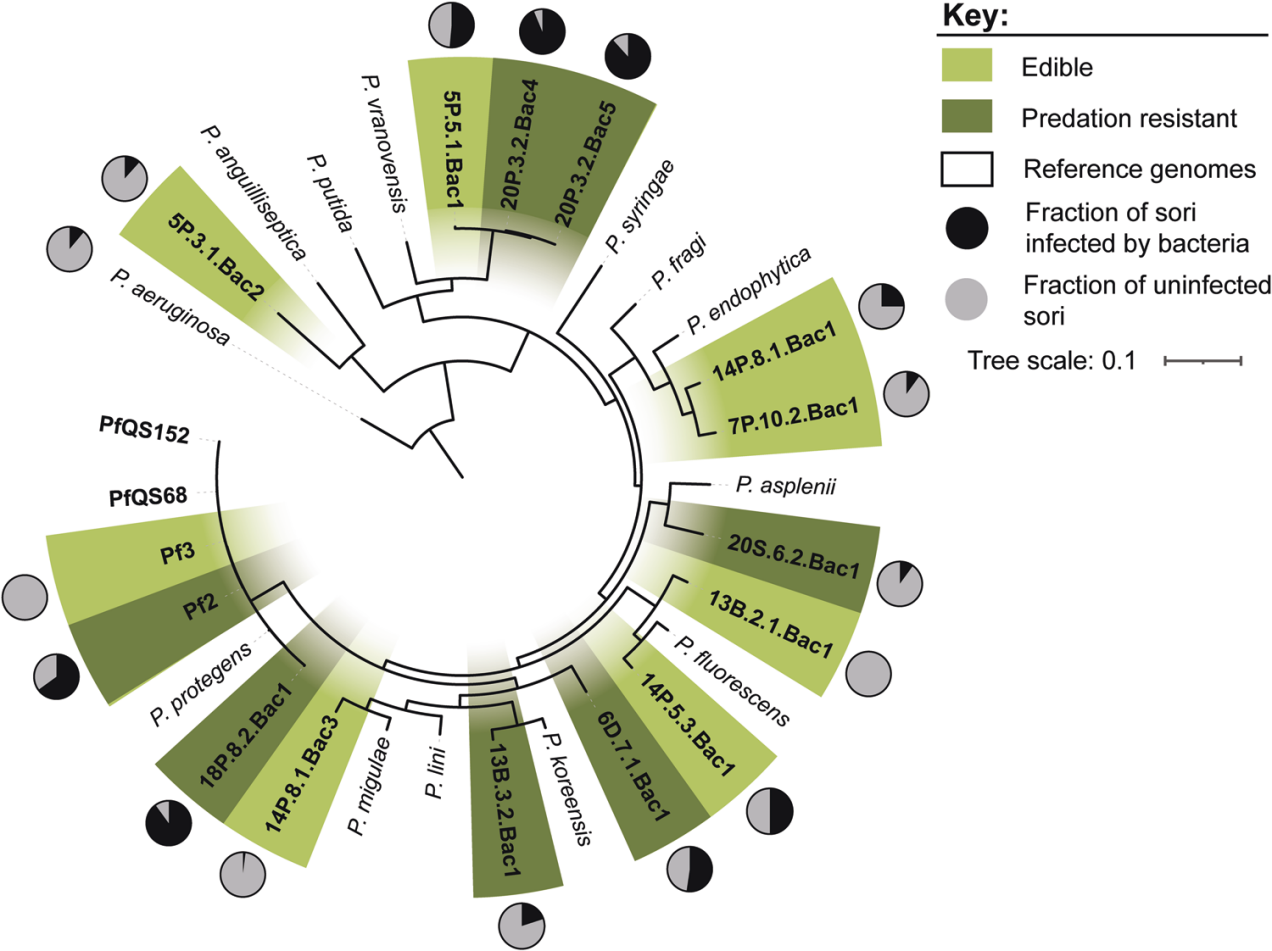
Diverse *Pseudomonas* strains evade predation and infect the sorus of *D. discoideum* fruiting bodies. A phylogeny, based on 1734 shared proteins, shows the evolutionary relationships between edible *Pseudomonas* isolates (light green), predation-resistant isolates (dark green), and reference genomes (white). Pie charts around the outer edge of the phylogeny show the fraction of sori that were infected with bacteria after *D. discoideum* was grown on a mixture of *Pseudomonas* and *K. pneumoniae*. 10–80 individual sori were sampled for each strain.

Some *Pseudomonas* strains are resistant to predation by *D. discoideum*, while others are susceptible [7]. However, it is not known how much diversity exists among predation-resistant strains or whether mechanisms of predation resistance have evolved more than once within the genus. To explore these questions, we selected fifteen *Pseudomonas* strains that were isolated from wild *D. discoideum* clones in two previous studies [7, 38]. Eight strains were susceptible to predation, meaning *D. discoideum* amoebae were able to clear agar plates of bacteria and form fruiting bodies even when no additional food bacterium was provided. The other seven strains were predation resistant, with large amounts of bacteria remaining on agar plates and few or no fruiting bodies after 7 d. We tested for the presence of bacteria in the sorus by collecting individual sori from fruiting bodies and transferring them to fresh agar plates. Because *D. discoideum* employs multiple mechanisms to eliminate bacteria during development to protect spores from potential pathogens, the sorus is typically expected to be bacteria-free. However, multiple *Pseudomonas* strains were able to infect the sorus (Fig. 1). We chose to focus on three predation-resistant strains. Strains 20P_3.2_Bac4, 20P_3.2_Bac5, and 18P_8.2_Bac1 infected more often than average (Fisher’s exact test, *p* < 0.05, FDR correction for multiple comparisons (Supplementary Table S2). However, 20P_3.2_Bac4 and 20P_3.2_Bac5 are very closely related, so we replaced the latter with the nearly significant 6D_7.1 Bac1, leaving us with 20P_3.2_Bac4, 18P_8.2_Bac1, and 6D_7.1_Bac1. Interestingly, the ability of 6D_7.1_Bac1 to infect the sorus is affected by temperature. When *D. discoideum*, 6D_7.1_Bac1, and *K. pneumoniae* are co-cultured at 18.6 °C, approximately half of the sori are infected by 6D_7.1_Bac1, while no sori are infected at 25 °C (Supplementary Table S3), so it is possible that fluctuations in laboratory temperature reduced the observed frequency of infection.

In most experiments, we grew *D. discoideum* on a mixture of *Pseudomonas* and food bacterium *K. pneumoniae* to allow amoebae to replicate and form fruiting bodies in the presence of inedible *Pseudomonas* strains. To rule out the possibility that the strains that resisted predation and infected the sorus were more successful because they are better at competing with *K. pneumoniae*, we compared the number of *Pseudomonas* CFU recovered from monoculture to the CFU recovered after coculture with *K. pneumoniae*. Growth of 5 edible strains and 4 predation-resistant strains was significantly reduced in the presence of *K. pneumoniae* (Supplementary Fig. S1A). However, this reduction is less than an order of magnitude for most strains and leaves more than 10^8^ *Pseudomonas* CFU on the plate. We used a Linear Mixed-effects Model to test the additive effects of treatment (monoculture or coculture with *K. pneumoniae*) and strain type (edible or predation-resistant) on log transformed CFU. We found that the fixed effect of treatment was significant (estimate = 1.2035, SE = 0.1558, *p* value = 3.01e−11), while the fixed effect strain type of was not significant (estimate = −1.4217, SE = 0.8678, *p* value = 0.124). The proportion of CFU recovered from coculture relative to monoculture did not differ significantly between edible and predation-resistant strains (Supplementary Fig. S1B). This suggests that competition with *K. pneumoniae* does not determine whether *Pseudomonas* evades predation or infects the sorus. Among the strains that infect the sorus, 18P_8.2_Bac1 and 20P_3.2_Bac4, which infect most frequently, reached higher abundances on plates than did 6D_7.1_Bac1, suggesting that the abundance of bacteria on plates may contribute to the frequency of infection. We also verified that GFP expression does not significantly reduce the growth rate of the *Pseudomonas* strains used in this study (Supplementary Fig. S2).

We sequenced and assembled the genomes of 13 *Pseudomonas* isolates (Supplementary Table S4). Two *Ps. protegens* genomes (Pf2 and Pf3) were previously sequenced. We constructed a phylogeny based on the 15 isolate genomes and reference genomes of related species (Fig. 1). Although all 15 strains belong to the *Pseudomonas fluorescens* complex, the predation-resistant strains are not monophyletic. We used Average Nucleotide Identity (ANI) to determine whether each isolate belongs to the same species as the reference genome with the most similar 16S rRNA gene sequence (Supplementary Table S5). For most isolates, ANI was less than 95% for their most closely related reference genome, suggesting they represent undescribed species.

### *Pseudomonas* infections of *D. discoideum* are extracellular

**Fig. 2:**
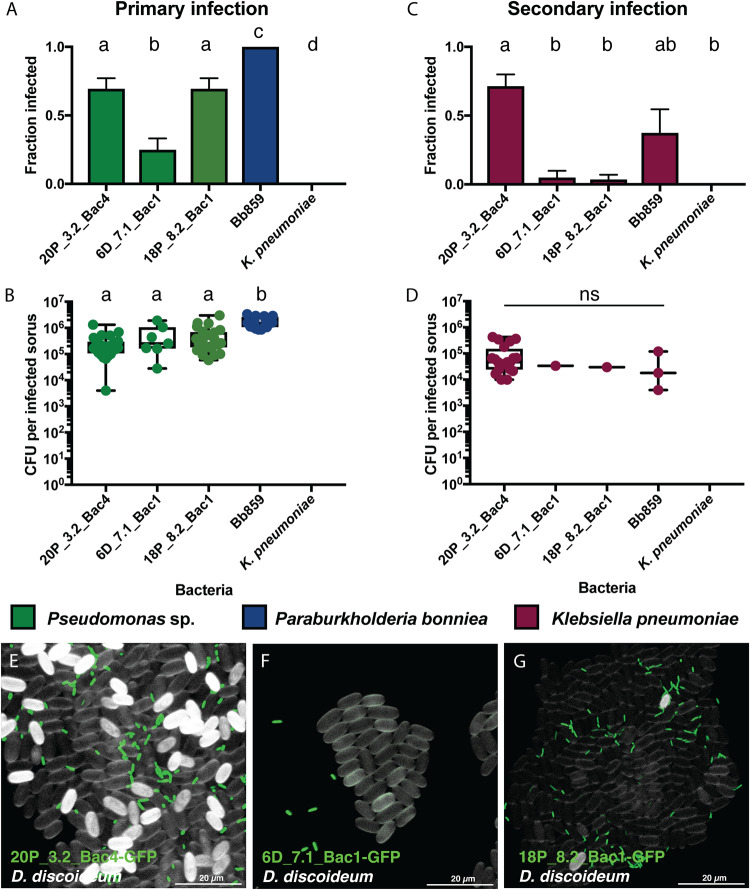
*Pseudomonas* infections within the sorus are extracellular. Sori were collected from *D. discoideum* fruiting bodies grown on mixtures of *K. pneumoniae*-E2crimson and GFP-labeled *Pseudomonas* spp., *Pa. bonniea* Bb859, or *K. pneumoniae*. Individual sori were placed in buffer, then serial dilutions were prepared and spotted on agar to quantify the number of GFP and E2crimson labeled bacteria per sorus. Some sori infected with *Pseudomonas* spp. or Bb859 also contained *K. pneumoniae*. **A**, **C** Fraction of sori infected with bacteria. Error bars show standard error. Fisher’s exact test was used to compare the number of infected and uninfected sori. Different letters indicate FDR corrected *p* ≤ 0.05. **B**, **D** Number of bacteria per infected sorus. Different letters indicate *p* ≤ 0.05, one-way ANOVA with Tukey’s multiple comparisons correction. Ns not significant. **A**, **B** Primary infection by *Pseudomonas* isolates or Bb859. **C**, **D** Secondary infection of the same sori by *K. pneumoniae*, which does not infect the sorus on its own. Microscopy images of spores and bacteria collected from sori infected with (**E**) 20P_3.2_Bac4-GFP, (**F**) 6D_7.1_Bac1-GFP, and (**G**) 18P_8.2_Bac1-GFP. Spore coats were stained with calcofluor, shown in white, while bacteria are shown in green.

To better understand how 20P_3.2_Bac4, 6D_7.1_Bac1, and 18P_8.2_Bac1 behave within the sorus, we quantified the number of bacteria per sorus in *D. discoideum* fruiting bodies grown on a mixture of *Pseudomonas* and *K. pneumoniae*. Although not all sori become infected by *Pseudomonas* (Fig. 2A), the number of bacteria within infected sori is consistent, suggesting that bacterial replication within the sorus may be restricted by limited space or nutrient availability (Fig. 2B). The number of *Pseudomonas* cells per sorus is also similar across species, with an average of 2.67 × 10^5^ 20P_3.2_Bac4, 5.74 × 10^5^ 6D_7.1_Bac1, and 5.41 × 10^5^ 18P_8.2_Bac1 bacteria per sorus. This is significantly less than the average number of *Pa. bonniea* Bb859 cells per sorus, 1.72 × 10^6^. *Pa. bonniea* is an intracellular symbiont that is present inside and outside of spores and has smaller cells and a reduced genome, which may help it replicate to higher densities within sori. As expected, *K. pneumoniae* does not infect the sorus on its own. Unlike other *Pseudomonas* species, the presence of 20P_3.2_Bac4 frequently allows secondary infections of the sorus by *K. pneumoniae* (Fig. 2C, D), a characteristic associated with symbiotic *Paraburkholderia*. Occasionally, sori of fruiting bodies grown with 20P_3.2_Bac4 contain only *K. pneumoniae*, suggesting 20P_3.2_Bac4 is not required for *K. pneumoniae* to survive within the sorus but instead has an effect earlier in development, possibly by inhibiting phagocytosis of *K. pneumoniae*.

We used fluorescence microscopy to determine whether bacteria were present inside or outside of spores. None of the three *Pseudomonas* species were found within spores, suggesting that infections within the sorus are extracellular (Fig. 2E–G). The bacteria appear to aggregate with clumps of spores, but cells were removed from the sorus and stained prior to imaging, so images may not accurately depict their spatial organization in situ.

**Fig. 3:**
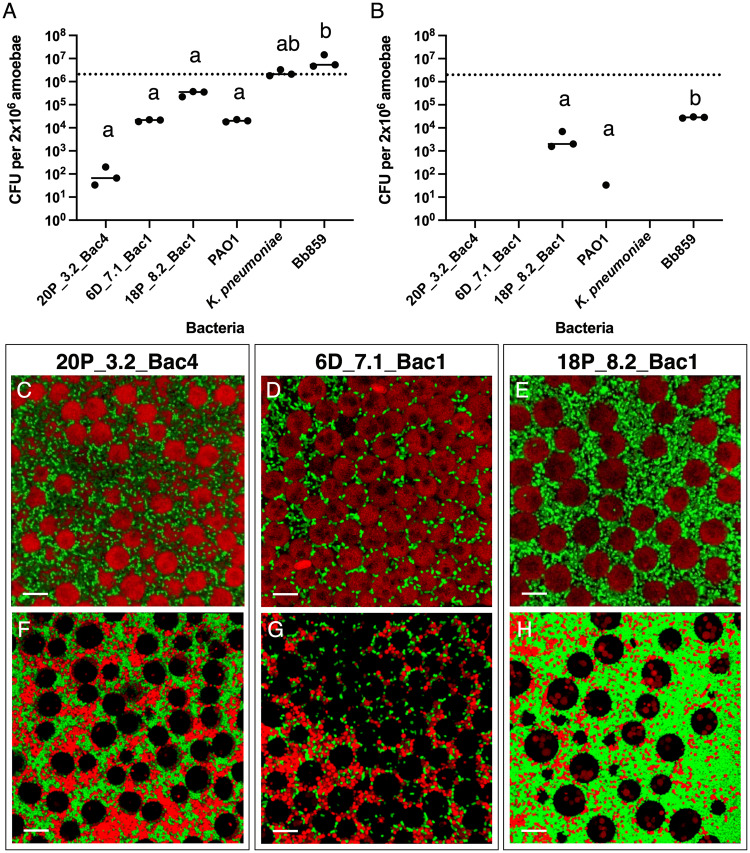
*Pseudomonas* sp. infections of amoebae are uncommon. Mixtures of *D. discoideum* AX4 amoebae and bacteria (*Pseudomonas* isolates 20P_3.2_Bac4, 6D_7.1_Bac1, and 18P_8.2_Bac1; pathogen *Ps. aeruginosa* PAO1; food bacterium *K. pneumoniae*; and intracellular symbiont *Pa. bonniea* Bb859) were treated with gentamicin to eliminate extracellular bacteria. After 3 h (**A**) or 22 h (**B**), amoebae were washed and lysed. Points show bacterial CFU recovered from lysate, while the dashed line represents the number of amoebae. Different letters indicate *p* ≤ 0.05, one-way ANOVA with Tukey’s multiple comparisons correction. Confocal microscopy was used to image bacteria and amoebae on soft agar to determine whether bacteria were located within amoebae. **C** 20P_3.2_Bac4-GFP, **D** 6D_7.1_Bac1-GFP, and **E** 18P_8.2_Bac1-GFP were grown with *D. discoideum* QS9.1-mCherry and unlabeled *K. pneumoniae* (**C**–**E**) or with *D. discoideum* QS9.1-mCherry and *K. pneumoniae*-E2crimson (**F**–**H**) on agar.

A hallmark of intracellular pathogens is the ability to infect and replicate within host cells. To determine whether 20P_3.2_Bac4, 6D_7.1_Bac1, or 18P_8.2_Bac1 replicate within amoebae, we cocultured each strain with axenically grown AX4 amoebae, added gentamicin to eliminate extracellular bacteria, then incubated the cells for 3 h or 22 h to determine how long each strain persisted intracellularly. We describe uptake of bacteria as ingestion and killing of bacteria within the phagosome as digestion. We included *Pseudomonas aeruginosa* PAO1, *K. pneumoniae*, and *Pa. bonniea* Bb859 for comparison, with the expectation that PAO1, a pathogen, and *Pa. bonniea*, an intracellular symbiont, would persist after phagocytosis, while *K. pneumoniae* would be rapidly taken up and eliminated. After 3 h of gentamicin treatment, we recovered relatively large numbers *K. pneumoniae* and *Pa. bonniea*; lower amounts of 6D_7.1_Bac1, 18P_8.2_Bac1, and PAO1; and very few 20P_3.2_Bac4 cells (Fig. 3). After 22 h, only 18P_8.2_Bac1, PAO1, and *Pa. bonniea* were recovered from amoebae, while the other species were below the limit of detection. For all *Pseudomonas* strains, the number of bacteria recovered was orders of magnitude lower than the initial number of amoebae. Two strains that infect the sorus, 6D_7.1_Bac1 and 18P_8.2_Bac1, appear to be taken up at a rate similar to the predation-resistant pathogen PAO1, while 20P_3.2_Bac4 is ingested even less frequently. Interestingly, 18P_8.2_Bac1 is taken up more frequently than PAO1 and demonstrates better survival within amoebae than any other strain except for the symbiotic *Pa. bonniea*. As expected, the food bacterium *K. pneumoniae* was taken up and digested, while intracellular symbiont *Pa. bonniea* was taken up and then survived within the amoebae. *Pa. bonniea* Bb859 is known to be slightly more edible than other symbiotic *Paraburkholderia* [39], which may explain the decrease in the number of intracellular bacteria over time.

To verify that predation-resistant *Pseudomonas* species are rarely taken up by *D. discoideum*, we used fluorescence microscopy to visualize interactions between mCherry-labeled QS9 amoebae and GFP-labeled bacteria on agar. After ~2 d coculture with GFP-labeled *Pseudomonas* and *K. pneumoniae*, the amoebae were embedded within a dense lawn of bacteria, but few bacteria were observed within amoebae (Fig. 3C–E). While it is possible to find amoebae that contain individual 6D_7.1_Bac1 and 18P_8.2_Bac1 cells, intracellular 20P_3.2_Bac4 is very uncommon, which is consistent with the results of the gentamicin protection assay. We also imaged mCherry-labeled QS9 amoebae with GFP-labeled *Pseudomonas* and E2crimson-labeled *K. pneumoniae* (Fig. 3F–H). *K. pneumoniae* is detectable inside of amoebae but, interestingly, large amounts of *K. pneumoniae* remain uneaten.

### Some predation-resistant *Pseudomonas* protect edible species

**Fig. 4:**
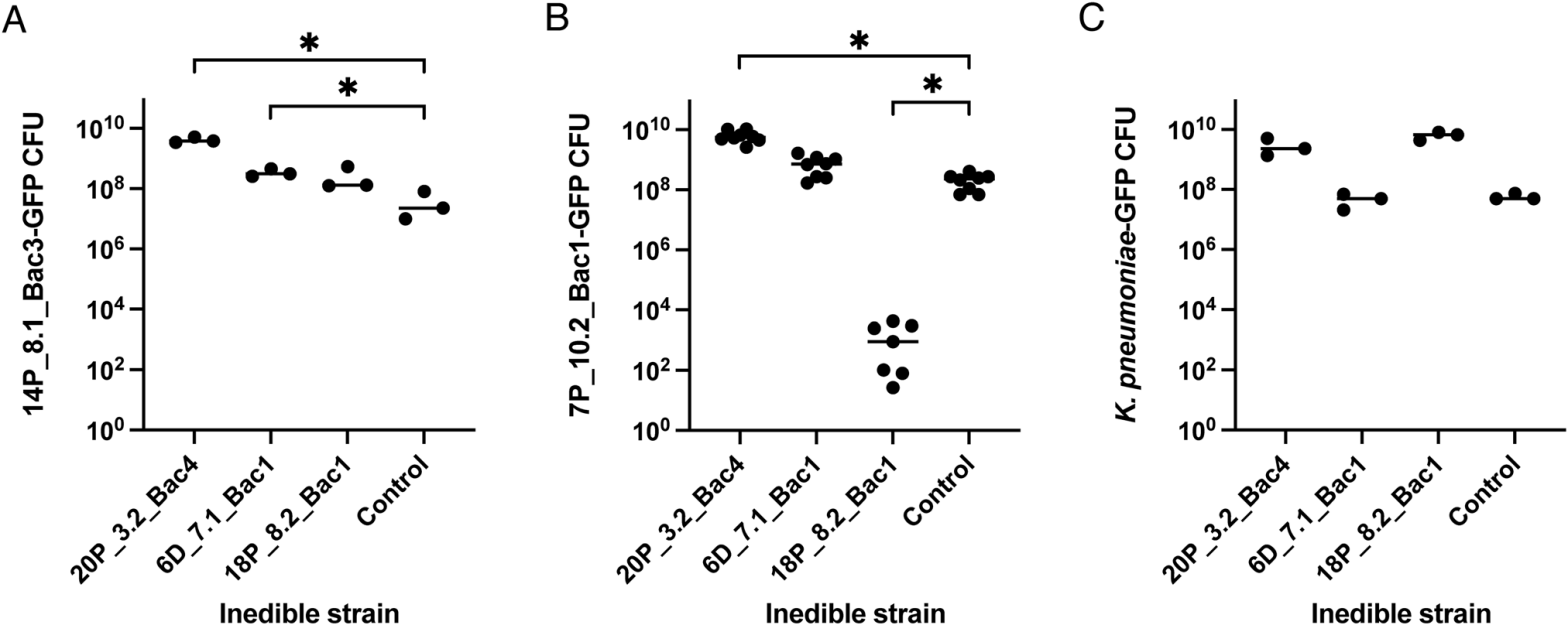
Some predation-resistant *Pseudomonas* strains protect edible bacteria from predation. **A** 14P_8.1_Bac3-GFP, **B** 7P_10.2_Bac1-GFP, and **C** *K. pneumoniae*-GFP CFUs recovered after 7 d co-culture with *D. discoideum* AX4. Edible bacteria were grown on SM/5 agar with a predation-resistant *Pseudomonas* strain (20P_3.2_Bac4, 6D_7.1_Bac1, or 18P_8.2_Bac1) and *D. discoideum* or with buffer and *D. discoideum* (control). Edible bacteria CFU were quantified by collecting cells from plates and spotting serial dilutions on selective media. Brown-Forsythe and Welch ANOVA with Dunnett’s T3 multiple comparisons test with individual variances comparing each sample to the control. *p ≤ 0.05.

Bacteria may evade predation by secreting proteins or metabolites that suppress predators. If 20P_3.2_Bac4, 6D_7.1_Bac1, or 18P_8.2_Bac1 use such a mechanism, we predicted that the presence of predation-resistant bacteria may benefit otherwise edible bacteria in co-culture with *D. discoideum*. We tested this hypothesis with *K. pneumoniae* and two edible *Pseudomonas* sp. strains: 14P_8.1_Bac3 and 7P_10.2_Bac1 (Fig. 4). The presence of 20P_3.2_Bac4 resulted in 110, 31, and 50-fold increases in 14P 8.1_Bac3, 7P 10.2_Bac1, and *K. pneumoniae* CFU recovered compared to when the edible bacteria were grown with AX4 and no predation-resistant bacteria, although the effect is only significant for 14P_8.1_Bac3 and 7P_10.2_Bac1. Coculture with 6D_7.1_Bac1 resulted in smaller 9 and 4-fold increases in 14P_ 8.1_Bac3 and 7P_10.2_Bac1 CFU recovered and did not protect *K. pneumoniae*. 18P_8.2_Bac1 increased the survival of *K. pneumoniae*-GFP 110-fold (not statistically significant) but dramatically reduced the survival of 7P_10.2_Bac1, suggesting antagonism between *Pseudomonas* strains.

### Diversity of potential predation resistance genes

**Fig. 5:**
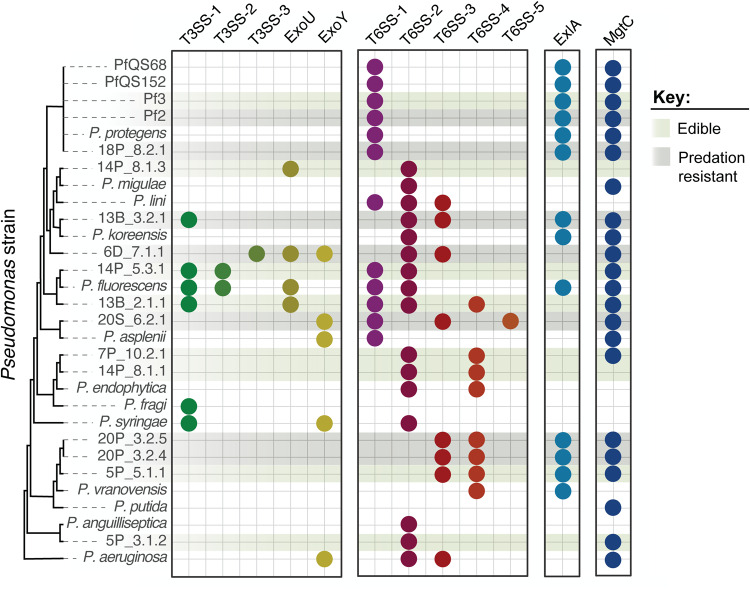
Presence of genes known to contribute to resistance to phagocytosis in other *Pseudomonas* species does not explain predation resistance in soil isolates. Colored circles represent homologs of T3SS structural genes, T3SS effectors ExoU and ExoY, T6SS structural genes, ExlA, and MgtC. Sequenced genomes (identified by isolate name) and reference genomes (identified by species name) are organized based on the whole genome phylogeny from Fig. 1. Light and dark shading highlights isolates that are edible or resistant to predation.

Several genes and gene clusters are known to help *Pseudomonas* sp. resist predation or survive within the phagosome. These genes include: Type III secretion systems (T3SS); T3SS effectors ExoU and ExoY; Type VI secretion systems (T6SS); ExlA, a pore forming toxin secreted by many *Pseudomonas* species; [40] and MgtC, which inhibits phagosome acidification and contributes to *Ps. aeruginosa* survival within macrophages [41, 42]. To determine whether the distribution of any of these genes could explain why some *Pseudomonas* species are resistant to predation by *D. discoideum*, while others are susceptible, we searched for homologs in the genomes we sequenced as well as related reference genomes (Fig. 5, Supplementary Table S6). We identified three different T3SS and five different T6SS. A few strains, including 6D 7.1_Bac1, have T3SS and encode homologs to T3SS effectors ExoU and ExoY. While almost all strains encode one or more T6SS, T6SS-3 is present in 5 of 7 predation-resistant strains and only 1 of 8 edible strains. Many strains encode homologs of ExlA and MgtC. However, none of these genes or gene clusters are found more frequently in predation-resistant strains than edible strains (Fisher’s exact test, *p* > 0.05, FDR correction for multiple comparisons) (Supplementary Table S7).

**Fig. 6:**
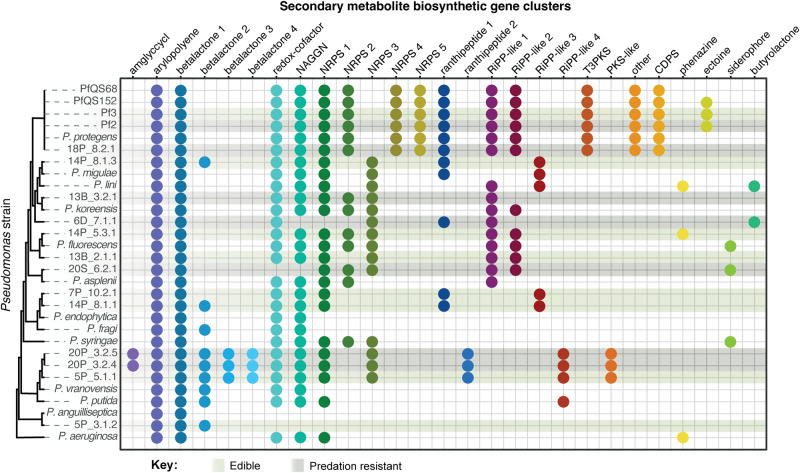
Distribution of secondary metabolite biosynthetic gene clusters in *Pseudomonas* genomes. Colored circles represent the presence of a cluster of biosynthetic genes. Clusters within the same group (x-axis labels) share >70% nucleotide identity over ≥20% of the cluster length. Sequenced genomes (identified by isolate name) and reference genomes (identified by species name) are organized based on the whole genome phylogeny from Fig. 1. Light and dark shading highlights isolates that are edible or resistant to predation.

Since secondary metabolites can also contribute to predation resistance, we used antiSMASH to identify putative secondary metabolite biosynthetic gene clusters, then grouped the gene clusters based on homology (Fig. 6, Supplementary Fig. S3). Some clusters were found in all or most *Pseudomonas* genomes, while others were limited to certain taxa. None of the secondary metabolite biosynthetic gene clusters are found more frequently in predation-resistant strains than in susceptible strains (Fisher’s exact test, *p* > 0.05, FDR correction for multiple comparisons) (Supplementary Table S7).

## Discussion

Multiple *Pseudomonas* species resist predation by *D. discoideum*, and some infect *D. discoideum* fruiting bodies. However, little is known about most *Pseudomonas* species that have been isolated from *D. discoideum*, many of which appear to belong to undescribed species based on low ANI with closely related NCBI reference genomes. Three isolates that infect *D. discoideum* fruiting bodies, though distantly related to each other, are each related to species associated with plant or animal hosts. 18P_8.2_Bac1 is a strain of *Ps. protegens*, a species that has been extensively studied in the context of biocontrol, as *Ps. protegens* strains produce a variety of secondary metabolites and exoproteins that suppress the growth of fungal and bacterial pathogens of plants [43]. Similarly, *Ps. lini*, a relative of 6D_7.1_Bac1, has been shown to suppress plant pathogens [44]. *Ps. vranovensis*, a relative of 20P_3.2_Bac4, is a pathogen of the nematode *Caenorhabditis elegans* [45], but no virulence mechanisms have been described.

Predation-resistant strains are not monophyletic, suggesting this trait has been gained or lost multiple times. A few proteins and protein complexes are known to contribute to resistance to phagocytosis or survival within the phagosome in other *Pseudomonas* species. For example, the human pathogen *Ps. aeruginosa* produces the pore-forming toxin Exolysin (ExlA) and secretes exotoxins ExoU and ExoY through a Type III secretion system (T3SS) to kill macrophages [40]. Among the predation-resistant *Pseudomonas* isolates identified in this study, only 6D_7.1_Bac1 encoded a T3SS and homologs to ExoU and ExoY. T3SSs were also present in edible strains 14P_5.3_Bac1 and 13B_2.1_Bac1, though these genes were distantly related to the T3SS of 6D_7.1_Bac1 and did not share synteny, suggesting the T3SSs are not closely related. *Ps. aeruginosa* also encodes MgtC-like proteins that contribute to virulence and survival within macrophages [41] and regulate expression of the T3SS [42]. In our study, ExlA and MgtC homologs were common among both edible and predation-resistant isolates, suggesting that the presence of these genes is not sufficient to confer predation resistance.

Some *Ps. protegens* strains rely on secondary metabolite production to escape from predation by *D. discoideum* and other protists [38, 46], so we also examined the secondary metabolite biosynthetic gene clusters that are encoded by our *Pseudomonas* isolates. Each predation-resistant isolate encoded multiple clusters, with little overlap. The same clusters are found in the predation-resistant 18P_8.2_Bac1 and Pf2 and edible Pf3 isolates, but Pf3 is known to be edible because of a nonsense mutation in *gacA*, which is part of the two-component system that regulates production of secondary metabolites [38]. 20P_3.2_Bac4 shares most of its clusters with the closely related isolate 5P_5.1_Bac1, which is categorized as edible but is not as good a food source as *K. pneumoniae*. 6D_7.1_Bac1 appears to be missing several clusters that are found in other species, and the clusters it encodes are shared with edible species. Overall, the presence and absence of any one of these genes or gene clusters cannot explain why some *Pseudomonas* strains are edible, while others are predation resistant. The predation-resistant strains we identified may use different mechanisms to evade predation. Alternatively, there may be shared predation resistance genes that have not yet been identified or genes that are present in both predation-resistant and susceptible strains may be regulated in different ways. Although the mechanism of predation resistance has not yet been identified, the observations that 20P_3.2_Bac4 protects edible *Pseudomonas* strains from predation, while 18P_8.2_Bac1 protects *K. pneumoniae*, are consistent with secretion of anti-predation molecule or protein that can benefit nearby cells. We did not observe as strong a protective effect from co-culture with 6D_7.1_Bac1, which may mean that it does not secrete predation resistance molecules or that the production of such molecules is temperature dependent, like its ability to infect the sorus. Convergence on a predation-resistant phenotype, even if it is through different mechanisms, emphasizes the influence of predation on the evolution of bacteria in soil communities.

Based on our microscopy and gentamicin protection assays, 20P_3.2_Bac4, 6D_7.1_Bac1, and 18P_8.2_Bac1 appear to be ingested less frequently than edible *K. pneumoniae* and symbiotic *Pa. bonniea*. We recovered substantial amounts of *K. pneumoniae* from amoebae lysed a few hours after antibiotic treatment, which is consistent with results of studies that use similar assays [23]. In our experiments, *D. discoideum* was able to digest the small amounts of 20P_3.2_Bac4 and 6D_7.1_Bac1 that it consumed, suggesting that the mechanism of predation resistance in these two species likely depends on not being taken up. Though 18P_8.2_Bac1 was able to persist after phagocytosis, the number of intracellular bacteria is small and decreases over time, suggesting 18P 8.2_Bac1 does not replicate intracellularly.

Interestingly, we found that at least one *Pseudomonas* species (20P_3.2_Bac4) can induce secondary infections of *D. discoideum* fruiting bodies by otherwise edible bacteria. This trait has previously been associated with three *Paraburkholderia* species that are intracellular symbionts of *D. discoideum* [13, 14, 39]. By inducing *D. discoideum* to carry or “farm” edible bacteria, symbiotic *Paraburkholderia* are beneficial to the host when spores disperse to areas where prey bacteria are scarce, even though they reduce spore production [15]. These *Paraburkholderia* species are intracellular and efficiently infect fruiting bodies, allowing them to disperse with spores and remain associated with *D. discoideum* over many generations. As a result, they likely experience selective pressure to minimize negative effects on the host, as vertically transmitted symbionts may be more likely to become mutualists rather than parasites [47]. *Pa. hayleyella* and *Pa. bonniea* demonstrate genome reduction [48], increased proportions of infected spores, and reduced numbers of bacteria per spore [49] when compared to *Pa. agricolaris*, which is consistent with adaptation to a symbiotic lifestyle. In contrast, *Pseudomonas* infections of fruiting bodies appear to be strictly extracellular and only some fruiting bodies become infected, which would naturally lead to a much less stable association between the bacteria and the host. However, bacteria that infect the sorus could still co-disperse with spores, potentially leading to selection for mutualistic or parasitic traits. Purely opportunistic interactions between bacteria and amoebae may be the evolutionary origin of more complex symbioses.

In conclusion, multiple environmental *Pseudomonas* species evade predation by *D. discoideum* and exhibit some traits characteristic of the *D. discoideum-Paraburkholderia* symbiosis. These symbiont-like behaviors, including infecting the sorus, persisting inside of amoebae, and inducing secondary infections, are likely byproducts of the mechanisms these bacteria use to promote their own survival when interacting with *D. discoideum* and other predators, rather than symbiotic adaptations. However, the presence of these traits in environmental bacteria suggests that the threshold for establishing symbiosis with *D. discoideum* may be low. The genes responsible for predation resistance in 20P_3.2_Bac4, 6D_7.1_Bac1, and 18P_8.2_Bac1 have not yet been identified, but it seems likely that predation resistance has evolved multiple times within *Pseudomonas* and may be achieved by multiple means.

### Supplementary information

Supplementary material

## Data Availability

16S rRNA gene sequences and genomes are available through Genbank (ON954494-ON954502 and JANLNW000000000-JANLOH000000000), while raw Illumina reads are available through the NCBI SRA (PRJNA857029). *Pseudomonas* and *D. discoideum* strains are available from the Queller/Strassmann lab upon request. Code is available at https://github.com/misteele/Dicty-Pseudomonas-genomes.
