# Supplementary material for: Predation-resistant Pseudomonas bacteria engage in symbiont-like behavior with the social amoeba Dictyostelium discoideum
Source: ISME J. 2023 Oct 26;17(12):2352–61. doi: 10.1038/s41396-023-01535-5 (PMC10689837; doi:10.1038/s41396-023-01535-5)
Supplement: Supplementary file 1 — Supplementary material [file 41396_2023_1535_MOESM1_ESM.docx]

**Supplementary material for “Predation-resistant *Pseudomonas* bacteria engage in symbiont-like behavior with the social amoeba *Dictyostelium discoideum*”**

Authors: Margaret I. Steele, Jessica Peiser, P. M. Shreenidhi, Joan E. Strassmann, David C. Queller

**Index for supplementary material:**

Supplementary methods p. 3-7

Culture conditions p. 3

Chromosomal insertion of fluorescent protein genes p. 3

Competition assay p. 5

16S rRNA gene sequencing p. 6

Genome sequencing and assembly p. 6

Converting OD_600_ measurements to CFU p. 7

| Figure S1. Competition with *K. pneumoniae* reduces the growth of some *Pseudomonas* strains but is not more likely to effect edible strains. | p. 11 |
| --- | --- |
| Figure S2. GFP expression does not reduce the growth rate of *Pseudomonas* isolates. | p. 12 |
| Figure S3. No secondary metabolite biosynthetic gene clusters are unique to predation-resistant *Pseudomonas* genomes. | p. 21 |
| Table S1. Strains and plasmids used in this study. | p. 8 |
| Table S2. Infected and uninfected sori collected from *D. discoideum* fruiting bodies grown on mixtures of *Pseudomonas* sp. and *K. pneumoniae*. | p. 14 |
| Table S3. Temperature affects the fraction of *D. discoideum* sori that become infected with 6D_7.1_Bac1. | p. 15 |
| Table S4. Genome assembly statistics. | p. 16 |
| Table S5. Average Nucleotide Identity between isolate genomes and the NCBI reference genome with the most similar 16S rRNA sequence. | p. 17 |
| Table S6. Amino acid percent identity shared between ExlA, ExoU, ExoY, and MgtC reference sequences and homologs from isolate genomes. | p. 18 |
| Table S7. Number of edible and predation-resistant genomes with and without putative predation-resistance genes. | p. 19 |
| References | p. 22 |

**Supplementary methods**

**Culture conditions**

Antibiotics and additives were used at the following concentrations, except where indicated: 20 µg/ml gentamicin (Gm), 30 µg/ml kanamycin (Km), 100 µg/ml carbenicillin (Crb), 300 µg/ml streptomycin (Str), 60 µg/ml spectinomycin (Spec), 20 µg/ml chloramphenicol (Cm), 100 µg/ml hygromycin, 0.3 mM 4,6-diaminopimelic acid (DAP). *Escherichia coli* strains used for conjugation were grown in LB broth with appropriate antibiotics at 37ºC, shaking at 225 rpm. Unless otherwise specified, *Klebsiella pneumoniae* and *Pseudomonas* spp. used in experiments were grown on LB (LB broth, Miller (Fisher)) plates overnight at 30ºC then collected and diluted to an OD_600_ of 1.5 in KK2 (2.25 g KH_2_PO_4_ (Sigma Aldrich) and 0.67 g K_2_HPO_4_ (Fisher Scientific) per L). OD_600_ to CFU conversions are provided in Table S1. For *D. discoideum* experiments on agar, 4x10^5^ spores and 200µl of a bacterial suspension were spread on SM/5 agar plates (2 g glucose (Fisher Scientific), 2 g bacteriological peptone (Oxoid), 2 g yeast extract (Oxoid), 0.2 g MgSO_4_•7H_2_O (Fisher Scientific), 1.9 g KH_2_PO_4_ (Sigma Aldrich), 1 g K_2_HPO (Fisher Scientific), and 15 g agar (Fisher Scientific) per L). *D. discoideum* cultures on SM/5 agar were incubated at room temperature (20-22ºC). Axenic *D. discoideum* cultures were incubated at 21ºC, shaking at 100 rpm in 20 ml HL5 medium including glucose (Formedium) supplemented with Crb and Str to prevent bacterial contamination.

**Chromosomal insertion of fluorescent protein genes**

Site-specific integration of *gfp* and antibiotic resistance markers into genomes of *Pseudomonas* isolates was achieved using the mini-Tn7 system, as previously described [1]. Briefly, *E. coli* MFD*pir*, a DAP auxotroph, was used to deliver plasmids carrying a transposon and transposase genes (see Table S1) to recipient cells through conjugation. Equal numbers of donor, helper, and recipient cells were mixed and spotted on LB DAP plates, then incubated at 30ºC overnight. Cells were collected, washed with KK2, and then spread on selective LB plates. *K. pneumoniae* was labeled with E2-crimson using the same method.

Growth curves were created to determine whether expression of GFP affected the growth of the labeled bacteria. Briefly, WT and GFP-labeled bacteria were grown on LB plates at 30ºC for 2 d. Cells were collected and suspended in KK2 at an OD of 1.5. 10 µl of the cell suspension was diluted in 990 µl of LB, then 100 µl volumes were transferred to 3 wells of a 96-well tissue culture plate. A Tecan Spark plate reader was used to measure the OD_600_ every hour for 24 h. Growthcurver [2] was then used the calculate the minimum doubling time for each strain.

The safe haven knock-in plasmid  pDM1514  was used to integrate a *mCherry* tag into the chromosome of  *D. discoideum* strain QS9 at the *act5* locus, as previously described [3]. Briefly, log phase QS9 amoebae were transformed with purified plasmid in 4mm gap electrocuvettes using an exponential delay protocol of two pulses of 500V, 4 ms duration separated by 2 seconds using a BioRad micropulser. Cells were diluted in 10ml SorMC (15 mM KH_2_PO_4_ (Sigma Aldrich), 2 mM Na_2_HPO_4_ (Sigma Aldrich), 50 µM MgCl_2_ (Sigma Aldrich), and 50 µM CaCl_2_ (Fisher)) with OD=2 Kp and hygromycin for selection. After 5 days, a colony was isolated and the knock-in was verified through PCR with primers P1.F (5’-GTAATTCAAGTGCACCATCAAAG-3’) and P2.R (5’-CCAGAAGGTGTTAAATCGTTG-3’) and gel electrophoresis. The wildtype QS9 showed an expected band size of approximately 2800 bp, while the transformant produced a 4200bp band, confirming the integration of the fluorescent tag and resistance cassette.

**Competition assay**

To determine whether *Pseudomonas* isolates differ in ability to compete with *K. pneumoniae*, GFP-labeled *Pseudomonas* strains were grown in monoculture or coculture with *K. pneumoniae* on SM/5 agar. Each strain was grown on LB agar for 2 d at 30ºC, then cells were collected and suspended in KK2 at an OD_600_ of 1.5. 25 µl of each *Pseudomonas* strain was mixed with either 225 µl *K. pneumoniae* or sterile buffer, then 65 µl of the mixture was spread on a 60 mm diameter SM/5 plates. Each treatment was performed in triplicate. After 7 d incubation at room temperature, cells were collected by flooding the plates with 5 ml KK2. Eleven 1:10 serial dilutions were prepared and 10 µl droplets of each dilution were spotted in triplicate on LB supplemented with Km or Gm. *Pseudomonas* CFU were counted after 1-2 d. For the few *Pseudomonas* isolates that could not be labeled with GFP, WT *Pseudomonas* was cocultured with *K. pneumoniae*-GFP and serial dilutions were spotted on non-selective agar.

We used a Linear Mixed-effects Model to determine whether edible *Pseudomonas* strains were more likely than predation-resistant strains to be affected by coculture with *K. pneumoniae.* We used *lmer* from the lmerTest package v3.1-3 [4, 5] in R v4.3.1 [6] to fit a model in which log transformed CFU was the response, treatment (coculture or monoculture) and type (edible or predation-resistant) were fixed factors, and strain was a random effect. To visualize the difference in competitive ability between edible and predation-resistant strains, we plotted the average CFU recovered from coculture/average CFU recovered from monoculture.

**16S rRNA gene sequencing**

Genomic DNA was extracted from bacteria using a Qiagen DNeasy blood and tissue kit. 10µl of DNase-free RNase (MilliporeSigma) was added to each sample after Proteinase K treatment to remove RNA. 16S ribosomal RNA genes of *Pseudomonas* soil isolates were sequenced to verify that all isolates belonged to genus *Pseudomonas* and to identify closely related reference genomes. 16S ribosomal RNA genes were amplified through PCR using universal 16S primers 27F (5’- AGAGTTTGATCCTGGCTCAG-3’) and 1507R (5’- TACCTTGTTACGACTTCACCCCAG-3’). We used a touchdown PCR program with an initial annealing temperature of 60ºC, which was reduced by 1ºC each cycle for the first 10 cycles, followed by 30 cycles with an annealing temperature of 50ºC. PCR products were purified and sequenced (Azenta Life Sciences). Related reference genomes were identified by using nucleotide BLAST [7] to search the NCBI 16S ribosomal RNA sequences database.

**Genome sequencing and assembly**

Genomes were sequenced by the Microbial Genome Sequencing Center (MiGS) using an Illumina NextSeq 2000 to generate 2x151 bp paired end reads. 1.3 to 1.7 million reads were obtained for each genome, providing 56 to 96x coverage. FastQC 0.11.9 [8] was used to check quality of reads. Reads were assembled using Unicycler v0.4.9 [9] and Quast 5.1.0rc1 [10] was used to assess the quality of the assemblies. CheckM v1.0.18 [11], implemented in KBase [12], was used to quantify genome completeness and contamination.

**Converting *Pseudomonas* and *K. pneumoniae* OD_600_ measurements to CFU**

*Pseudomonas* strains and *K. pneumoniae* were grown on LB agar for 2 d at 30ºC. Cells were collected and suspended in KK2. Each suspension was diluted to approximately OD 1 and OD 0.1. The OD_600_ of each dilution was measured and recorded. Seven 1:10 serial dilutions were prepared for each suspension and 10 µl droplets of each dilution were spotted on LB agar in triplicate. CFU were counted after 1-2 d and used to calculate the original CFU per 1 ml cell suspension. The measured OD_600_ was plotted against CFU and a linear regression was fitted to the data. The slope of the line was used to estimate the CFU per 1 ml of a cell suspension with an OD_600_ of 1.5, which is the initial concentration of cells used to set up most experiments.

**Table S1. Strains and plasmids used in this study.**

| Species | Strain | Description | Source | CFU per 1 ml of OD_600_ 1.5 |
| --- | --- | --- | --- | --- |
| *Escherichia coli* | MFD*pir* | Donor strain for conjugations |  |  |
|  | WM3064 | Donor strain for conjugations | [13] |  |
| *Klebsiella pneumoniae* | Kp |  | (Dictybase stock center) | 2.06x10^9^ |
|  | Kp-GFP | Chromosomal insertion of GmR and GFP at attTn7 site | (this study) |  |
|  | Kp-E2crimson | Chromosomal insertion of GmR and E2crimson at attTn7 site | (this study) |  |
| *Paraburkholderia bonniea* | Bb859 | Isolate |  |  |
|  | Bb859-GFP | Chromosomal insertion of GmR and GFP at attTn7 site | (this study) |  |
| *Pseudomonas aeruginosa* | PAO1 |  |  | 9.31x10^8^ |
|  | PAO1-GFP | Chromosomal insertion of GmR and GFP at attTn7 site | [14] |  |
| *Pseudomonas* sp. | 5P_3.1_Bac2 | Isolate | [15] | 3.62x10^9^ |
|  | 5P_5.1_Bac1 | Isolate | [15] | 6.89x10^8^ |
|  | 5P_5.1_Bac1-GFP | Chromosomal insertion of KmR and GFP at attTn7 site | (this study) |  |
|  | 6D_7.1_Bac1 | Isolate | [15] | 1.24x10^9^ |
|  | 6D_7.1_Bac1-GFP | Chromosomal insertion of GmR and GFP at attTn7 site | (this study) |  |
|  | 7P_10.2_Bac1 | Isolate | [15] | 1.33x10^9^ |
|  | 7P_10.2_Bac1-GFP | Chromosomal insertion of KmR and GFP at attTn7 site | (this study) |  |
|  | 13B_2.1_Bac1 | Isolate | [15] | 9.68 x10^8^ |
|  | 13B_3.2_Bac1 | Isolate | [15] | 4.64x10^8^ |
|  | 13B_3.2_Bac1-GFP | Chromosomal insertion of GmR and GFP at attTn7 site | (this study) |  |
|  | 14P_5.3_Bac1 | Isolate | [15] | 1.52x10^9^ |
|  | 14P_5.3_Bac1-GFP | Chromosomal insertion of GmR and GFP at attTn7 site | (this study) |  |
|  | 14P_8.1_Bac1 | Isolate | [15] | 1.91x10^9^ |
|  | 14P_8.1_Bac3 | Isolate | [15] | 7.77x10^8^ |
|  | 14P_8.1_Bac3-GFP | Chromosomal insertion of GmR and GFP at attTn7 site | (this study) |  |
|  | 20P_3.2_Bac4 | Isolate | [15] | 8.26x10^8^ |
|  | 20P_3.2_Bac4-GFP | Chromosomal insertion of KmR and GFP at attTn7 site | (this study) |  |
|  | 20P_3.2_Bac5 | Isolate | [15] | 6.65x10^8^ |
|  | 20P_3.2_Bac4-GFP | Chromosomal insertion of GmR and GFP at attTn7 site | (this study) |  |
|  | 20S_6.2_Bac1 | Isolate | [15] | 7.19x10^8^ |
|  | 20S_6.2_Bac1-GFP | Chromosomal insertion of GmR and GFP at attTn7 site | (this study) |  |
| *Pseudomonas protegens* | 18P_8.2_Bac1 | Isolate | [15] | 1.52x10^9^ |
|  | 18P_8.2_Bac1-GFP | Chromosomal insertion of GmR and GFP at attTn7 site | (this study) |  |
|  | Pf2 | Isolate | [16] | 9.26x10^8^ |
|  | Pf3 | Isolate | [16] | 9.45x10^8^ |
| *Dictyostelium discoideum* | AX4 | Axenic clone | (Dictybase stock center) |  |
|  | QS157 | Wild clone | [16] |  |
|  | QS9 | Wild clone | [16] |  |
|  | QS9-mCherry | Wild clone with chromosomal mCherry integration at act5 locus | (this study) |  |
|  |  |  |  |  |
|  | Plasmid | Description | Source |  |
|  | pTNS2 | Tn7 transposase | [1] |  |
|  | pUC18R6KT-miniTn7T-Gm-sfGFP | mini-Tn7 transposon carrying GmR and GFP | [17] |  |
|  | pUC16R6KT-miniTn7-Gm-PA3-E2crimson | mini-Tn7 transposon carrying GmR and E2crimson | Julie Perreau |  |
|  | pURR25 | mini-Tn7 transposon carrying KmR and GFP | [13] |  |
|  | pUX-BF13 | Tn7 transposase | [13] |  |
|  | pDM1514 | Plasmid for knock-in of mCherry at act5 locus in *D. discoideum* | [3]  Addgene # 108999 |  |

**
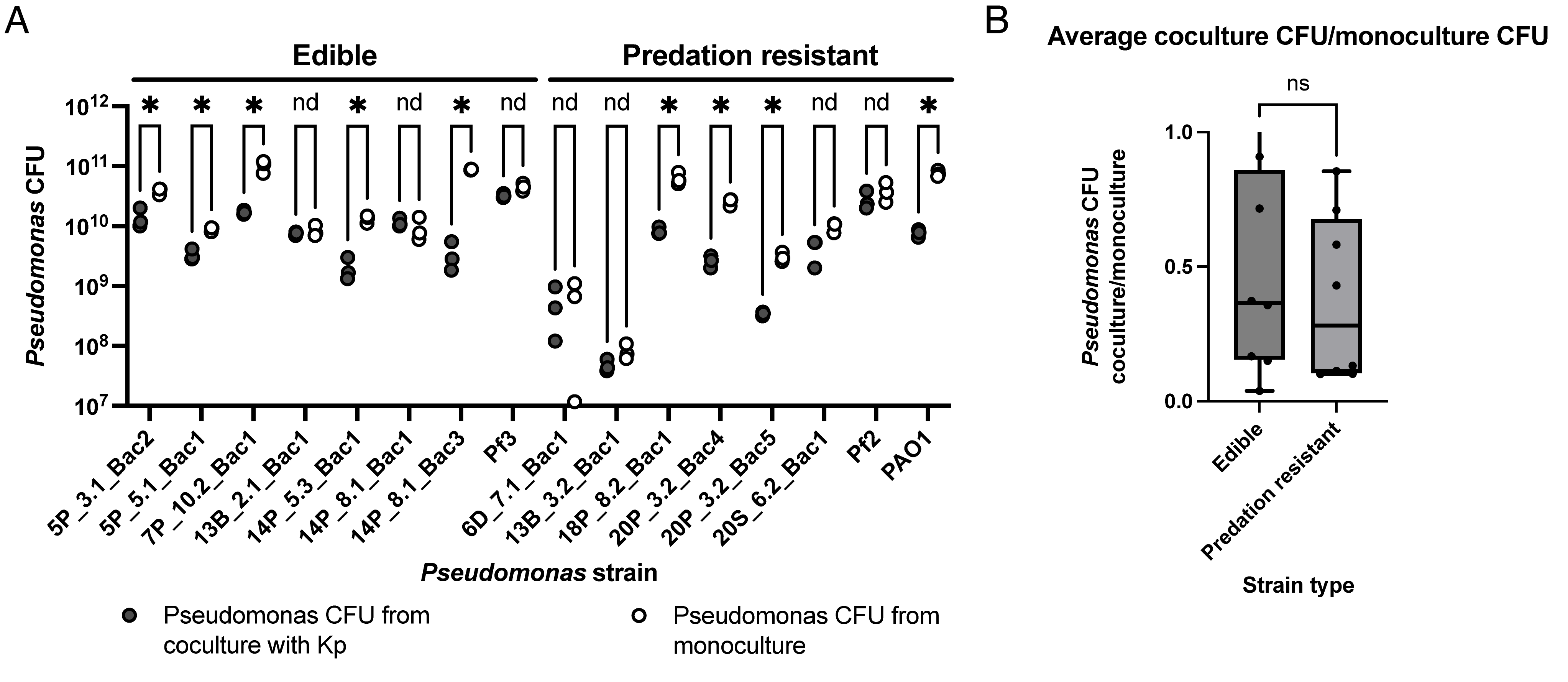
**

**Figure S1. Competition with *K. pneumoniae* reduces the growth of some *Pseudomonas* strains but is not more likely to effect edible strains.** (A) *Pseudomonas* CFU recovered from monoculture (white) or coculture with *K. pneumoniae* (grey). Unpaired t-test with two-stage linear step-up procedure of Benjamini, Krieger, and Yekutieli to correct for multiple comparisons. *, FDR < 1%; nd, not a discovery. Edible isolates are on the left, predation-resistant isolates on the right. Each point is an independent replicate. (B) The proportion of *Pseudomonas* CFU recovered from coculture relative to monoculture for edible and predation-resistant isolates. For each strain, the average CFU recovered from three coculture plates was divided by the average CFU recovered from three monoculture plates. Each point represents one strain. Welch’s t-test. ns, not significant.

**
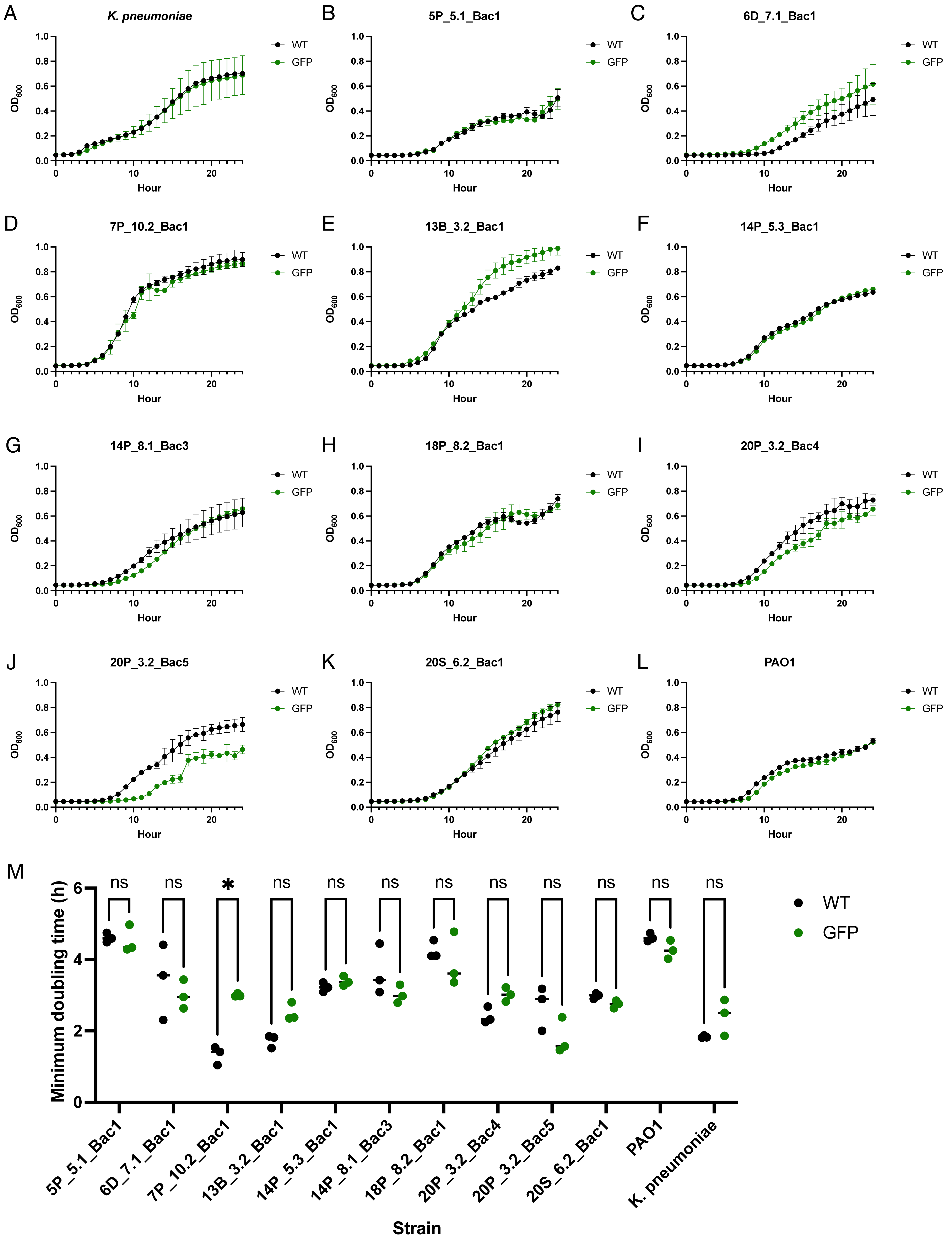
**

**Figure S2. GFP expression does not reduce the growth rate of *Pseudomonas* isolates.** Growth curves for WT and GFP-labeled bacteria (A) K. pneumoniae, (B) 5P_5.1_Bac1, (C) 6D_7.1_Bac1, (D) 7P_10.2_Bac1, (E) 13B_3.2_Bac1, (F) 14P_5.3_Bac1, (G) 14P_8.1_Bac3, (H) 18P_8.2_Bac1, (I) 20P_3.2_Bac4, (J) 20P_3.2_Bac5, (K) 20S_6.2_Bac1, and (L) PAO1. (M) Minimum doubling times for each strain. Two-way ANOVA with Sidak’s multiple comparisons test with a single pooled variance, *, adjusted p value < 0.05; ns, no significant difference.

**Table S2. The number of infected and uninfected sori collected from *D. discoideum* fruiting bodies grown on mixtures of *Pseudomonas* sp. and *K. pneumoniae*.** Fisher’s exact test was used to compare the number of infected and uninfected sori for each strain to the total number of infected and uninfected sori. False discovery rate (FDR) was used to correct p values for multiple comparisons.

| **Strain** | **Strain edibility** | **Infected sori** | **Uninfected sori** | **FDR adjusted pvalue** |
| --- | --- | --- | --- | --- |
| **Pf3** | Edible | 0 | 60 | 2.14E-12 |
| **14P_8.1_Bac3** | Edible | 1 | 49 | 6.42E-09 |
| **13B_2.1_Bac1** | Edible | 0 | 50 | 1.89E-10 |
| **7P_10.2_Bac1** | Edible | 5 | 45 | 2.47E-05 |
| **14P_8.1_Bac1** | Edible | 10 | 30 | 1.11E-01 |
| **5P_5.1_Bac1** | Edible | 36 | 34 | 7.31E-02 |
| **5P_3.1_Bac2** | Edible | 7 | 53 | 1.27E-05 |
| **14P_5.3_Bac1** | Edible | 5 | 5 | 5.28E-01 |
| **Pf2** | Inedible | 13 | 7 | 5.35E-02 |
| **18P_8.2_Bac1** | Inedible | 18 | 2 | 1.27E-05 |
| **6D_7.1_Bac1** | Inedible | 34 | 31 | 6.78E-02 |
| **20S_6.2_Bac1** | Inedible | 1 | 9 | 1.11E-01 |
| **20P_3.2_Bac4** | Inedible | 75 | 5 | 1.66E-21 |
| **20P_3.2_Bac5** | Inedible | 68 | 9 | 1.74E-16 |
| **13B_3.2_Bac1** | Inedible | 2 | 8 | 3.51E-01 |
| **PAO1** | Inedible | 4 | 32 | 5.91E-04 |

**Table S3.** **Temperature affects the fraction of *D. discoideum* sori that become infected with 6D_7.1_Bac1.** No fruiting bodies developed on 90% 6D_7.1_Bac1 plates incubated at 18ºC.

|  | | 18ºC incubation | | 25ºC incubation | |
| --- | --- | --- | --- | --- | --- |
| Treatment | Replicate | Sori with bacteria | Total sori tested | Sori with bacteria | Total sori tested |
| 10% 6D_7.1_Bac1 | 1 | 5 | 10 | 0 | 10 |
|  | 2 | 5 | 10 | 0 | 10 |
| 50% 6D_7.1_Bac1 | 1 | 2 | 5 | 0 | 3 |
|  | 2 | 5 | 8 | 0 | 10 |
| 90% 6D_7.1_Bac1 | 1 |  | 0 | 0 | 1 |
|  | 2 |  | 0 | 0 | 3 |

**Table S4. Genome assembly statistics.** CheckM was used to estimate genome completeness and contamination by searching for single-copy genes expected to be present in all strains.

| **Isolate** | **Contigs** | **Length (Mb)** | **N50** | **Coverage** | **%GC** | **Completeness** | **Contamination** |
| --- | --- | --- | --- | --- | --- | --- | --- |
| 5P_3.1_Bac2 | 38 | 4.74 | 556490 | 92.4 | 59.0 | 98.43 | 0.27 |
| 5P_5.1_Bac1 | 134 | 6.55 | 154624 | 78.0 | 63.3 | 99.92 | 0.82 |
| 6D_7.1_Bac1 | 88 | 6.05 | 160159 | 72.2 | 59.0 | 99.03 | 0.67 |
| 7P_10.2_Bac1 | 118 | 5.42 | 120580 | 96.2 | 57.7 | 100 | 0.58 |
| 13B_2.1_Bac1 | 131 | 6.85 | 137022 | 64.7 | 61.6 | 100 | 1.41 |
| 13B_3.2_Bac1 | 167 | 6.15 | 70603 | 67.1 | 60.4 | 100 | 0.11 |
| 14P_5.3_Bac1 | 98 | 6.31 | 171213 | 74.3 | 60.6 | 100 | 0.25 |
| 14P_8.1_Bac1 | 133 | 5.56 | 89697 | 73.7 | 58.3 | 99.03 | 0.62 |
| 14P­_8.1_Bac3 | 84 | 6.52 | 179469 | 68.5 | 60.5 | 99.35 | 1.22 |
| 20P_3.2_Bac4 | 51 | 6.81 | 400810 | 69.5 | 63.4 | 98.7 | 0.84 |
| 20P_3.2_Bac5 | 173 | 6.53 | 100119 | 65.6 | 63.3 | 99.88 | 1.18 |
| 20S_6.2_Bac1 | 164 | 6.53 | 108314 | 74.1 | 63.3 | 99.92 | 1.34 |
| 18P 8.2_Bac1 | 98 | 7.46 | 209371 | 56.5 | 61.4 | 99.02 | 0.77 |

**Table S5.** **Average Nucleotide Identity between isolate genomes and the NCBI reference genome with the most similar 16S rRNA sequence.** 18P_8.2_Bac1 is closely related to *Pseudomonas* *protegens* CHA0 (98.8% ANI). 20P_3.2_Bac4 and 20P 3.2_Bac5, which are nearly identical (99.99% ANI), are most closely related to *Pseudomonas* *alkylphenolica* (84.5 and 84.6% ANI) but were identified as *Pseudomonas* *vranovensis* (84.3 and 84.4% ANI) when they were first isolated. 6D_7.1_Bac1 was originally identified as *Pseudomonas* *lini* (85.7% ANI) but is more closely related to *Pseudomonas* *frederiksbergensis* (85.9% ANI).

| Isolate | Reference genome | Reference accession | ANI |
| --- | --- | --- | --- |
| 5P_3.1_Bac2 | *Pseudomonas anguilliseptica* | GCF_900105355.1 | 81.48 |
| 5P_5.1_Bac1 | *Pseudomonas alkylphenolica* | GCF_009755645.1 | 84.54 |
| 6D_7.1_Bac1 | *Pseudomonas frederiksbergensis* | GCF_002967995.1 | 85.88 |
| 7P_10.2_Bac1 | *Pseudomonas helleri* | GCF_001043025.1 | 89.03 |
| 13B_2.1_Bac1 | *Pseudomonas lurida* | GCF_001708485.1 | 89.08 |
| 13B_3.2_Bac1 | *Pseudomonas koreensis* | GCF_900101415.1 | 91.96 |
| 14P_5.3_Bac1 | *Pseudomonas fluorescens* | GCF_900215245.1 | 89.51 |
| 14P_8.1_Bac1 | *Pseudomonas helleri* | GCF_001043025.1 | 96.48 |
| 14P_8.1_Bac3 | *Pseudomonas migulae* | GCF_900106025.1 | 89.37 |
| 20P_3.2_Bac4 | *Pseudomonas alkylphenolica* | GCF_009755645.1 | 84.52 |
| 20P_3.2_Bac5 | *Pseudomonas alkylphenolica* | GCF_009755645.1 | 84.61 |
| 20S_6.2_Bac1 | *Pseudomonas asplenii* | GCF_900105475.1 | 85.18 |
| 18P 8.2_Bac1 | *Pseudomonas protegens* | GCF_900560965.1 | 98.77 |
| Pf2 | *Pseudomonas protegens* | GCF_900560965.1 | 98.77 |
| Pf3 | *Pseudomonas protegens* | GCF_900560965.1 | 98.73 |
| PfQS152 | *Pseudomonas protegens* | GCF_900560965.1 | 98.77 |
| PfQS68 | *Pseudomonas protegens* | GCF_900560965.1 | 98.82 |

**Table S6.** **Amino acid percent identity shared between ExlA, ExoU, ExoY, and MgtC reference sequences and homologs from isolate genomes.**

| **Strain** | **ExlA** | **ExoU** | **ExoY** | **MgtC** |
| --- | --- | --- | --- | --- |
| *Pseudomonas aeruginosa* |  |  | 99.74 | 100 |
| *Pseudomonas* sp. 5P_3.1_Bac2 |  |  |  | 46.52 |
| *Pseudomonas anguilliseptica* |  |  |  |  |
| *Pseudomonas putida* |  |  |  | 86.92 |
| *Pseudomonas vranovensis* | 39.16 |  |  |  |
| *Pseudomonas* sp. 5P_5.1_Bac1 | 39.73 |  |  | 87.61 |
| *Pseudomonas* sp. 20P_3.2_Bac4 | 39.51 |  |  | 88.03 |
| *Pseudomonas* sp. 20P_3.2_Bac5 | 39.51 |  |  | 88.03 |
| *Pseudomonas syringae* |  |  | 24.61 |  |
| *Pseudomonas fragi* | 51.42 |  |  |  |
| *Pseudomonas endophytica* |  |  |  |  |
| *Pseudomonas* sp. 14P_8.1_Bac1 |  |  |  |  |
| *Pseudomonas* sp. 7P_10.2_Bac1 |  |  |  | 44.2 |
| *Pseudomonas asplenii* |  |  | 27.93 | 45.25 |
| *Pseudomonas* sp. 20S_6.2_Bac1 |  |  | 30.58 | 44.55 |
| *Pseudomonas* sp. 13B_2.1_Bac1 | 56.65 | 43.36 |  | 43.81 |
| *Pseudomonas fluorescens* | 59.4 | 45.46 |  | 44.25 |
| *Pseudomonas* sp. 14P_5.3_Bac1 |  |  |  | 43.81 |
| *Pseudomonas* sp. 6D_7.1_Bac1 |  | 62.94 | 33.69 | 43.11 |
| *Pseudomonas koreensis* | 56.5 |  |  | 44 |
| *Pseudomonas* sp. 13B_3.2_Bac1 |  |  |  | 44 |
| *Pseudomonas lini* |  |  |  |  |
| *Pseudomonas migulae* |  |  |  | 44.89 |
| *Pseudomonas* sp. 14P_8.1_Bac3 |  | 45.19 |  |  |
| *Pseudomonas protegens* 18P_8.2_Bac1 | 59.35 |  |  | 44.25 |
| *Pseudomonas protegens* | 59.41 |  |  | 44.25 |
| *Pseusomonas fluorescens* Pf2 | 59.35 |  |  | 44.25 |
| *Pseusomonas fluorescens* Pf3 | 59.35 |  |  | 44.25 |
| *Pseusomonas fluorescens* PfQS152 | 59.35 |  |  | 44.25 |
| *Pseusomonas fluorescens* PfQS68 | 59.27 |  |  | 44.69 |

**Table S7. Number of edible and predation-resistant genomes with and without putative predation-resistance genes.** For each gene or complex, Fisher’s exact test was used to compare the number of edible strains with and without the gene to the number of inedible strains with and without the gene. FDR corrected p values are shown.

| Gene(s) | Edible strains with gene | Edible strains without gene | Predation resistant strains with gene | Predation resistant strains without gene | p value | FDR corrected p value |
| --- | --- | --- | --- | --- | --- | --- |
| T3SS-1 | 2 | 6 | 1 | 6 | 1.00 | 1.00 |
| T3SS-2 | 1 | 7 | 0 | 7 | 1.00 | 1.00 |
| T3SS-3 | 0 | 8 | 1 | 6 | 0.47 | 0.83 |
| ExoU | 2 | 6 | 1 | 6 | 1.00 | 1.00 |
| ExoY | 0 | 8 | 2 | 5 | 0.20 | 0.83 |
| T6SS-1 | 3 | 5 | 3 | 4 | 1.00 | 1.00 |
| T6SS-2 | 6 | 2 | 2 | 5 | 0.13 | 0.83 |
| T6SS-3 | 1 | 7 | 5 | 2 | 0.04 | 0.83 |
| T6SS-4 | 4 | 4 | 2 | 5 | 0.61 | 0.83 |
| T6SS-5 | 0 | 8 | 1 | 6 | 0.47 | 0.83 |
| ExlA | 2 | 6 | 5 | 2 | 0.13 | 0.83 |
| MgtC | 6 | 2 | 7 | 0 | 0.47 | 0.83 |
| Amglyccycl | 0 | 8 | 2 | 5 | 0.20 | 0.83 |
| Betalactone 1 | 4 | 4 | 2 | 5 | 0.61 | 0.83 |
| Betalactone 3 | 1 | 7 | 2 | 5 | 0.57 | 0.83 |
| Betalactone 4 | 1 | 7 | 2 | 5 | 0.57 | 0.83 |
| Redox-cofactor | 7 | 1 | 6 | 1 | 1.00 | 1.00 |
| NAGGN | 7 | 1 | 5 | 2 | 0.57 | 0.83 |
| NRPS 1 | 7 | 1 | 6 | 1 | 1.00 | 1.00 |
| NRPS 2 | 2 | 6 | 4 | 3 | 0.31 | 0.83 |
| NRPS-like 3 | 4 | 4 | 5 | 2 | 0.61 | 0.83 |
| NRPS 4 | 1 | 7 | 2 | 5 | 0.57 | 0.83 |
| NRPS 5 | 1 | 7 | 2 | 5 | 0.57 | 0.83 |
| Ranthipeptide 1 | 4 | 4 | 3 | 4 | 1.00 | 1.00 |
| Ranthipeptide 2 | 1 | 7 | 2 | 5 | 0.57 | 0.83 |
| RiPP-like 1 | 3 | 5 | 5 | 2 | 0.31 | 0.83 |
| RiPP-like 2 | 3 | 5 | 3 | 4 | 1.00 | 1.00 |
| RiPP-like 3 | 3 | 5 | 0 | 7 | 0.20 | 0.83 |
| RiPP-like 4 | 1 | 7 | 2 | 5 | 0.57 | 0.83 |
| T3PKS | 1 | 7 | 2 | 5 | 0.57 | 0.83 |
| PKS-like | 1 | 7 | 2 | 5 | 0.57 | 0.83 |
| Other | 1 | 7 | 2 | 5 | 0.57 | 0.83 |
| CDPS | 1 | 7 | 2 | 5 | 0.57 | 0.83 |
| Phenazine | 1 | 7 | 0 | 7 | 1.00 | 1.00 |
| Ectoine | 1 | 7 | 1 | 6 | 1.00 | 1.00 |
| Siderophore | 0 | 8 | 1 | 6 | 0.47 | 0.83 |
| Butyrolactone | 0 | 8 | 1 | 6 | 0.47 | 0.83 |


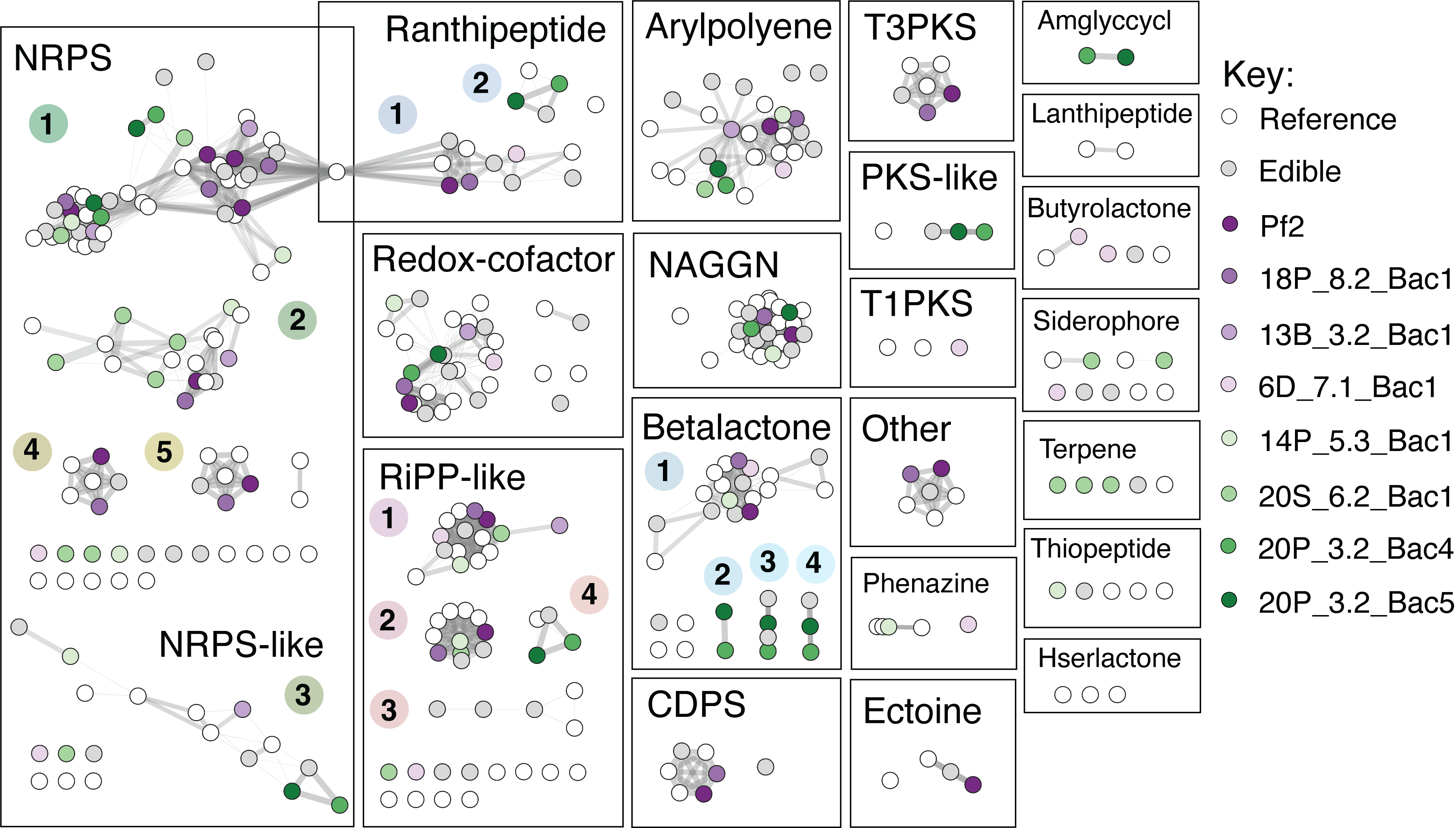


**Figure S3. No secondary metabolite biosynthetic gene clusters are unique to predation-resistant *Pseudomonas* genomes.** Homology network diagram showing relationships between secondary metabolite gene clusters in *Pseudomonas* genomes. Nodes represent nucleotide sequences of gene clusters. Nodes representing sequences from predation-resistant isolates are colored according to species. Grey nodes represent sequences from edible isolates, while white nodes are sequences from reference genomes. Lines connect nodes that share >70% nucleotide identity over more than 20% of the total length of the cluster. Line color represents percent identity, while width is proportional to alignment length relative to query length. Clusters are numbered when more than one cluster of a kind was identified.

**References**

1. Choi K-H, Schweizer HP. mini-Tn*7* insertion in bacteria with single *att*Tn*7* sites: example *Pseudomonas aeruginosa*. *Nat Protoc* 2006; **1**: 153–161.

2. Sprouffske K, Wagner A. Growthcurver: an R package for obtaining interpretable metrics from microbial growth curves. *BMC Bioinformatics* 2016; **17**: 172.

3. Paschke P, Knecht DA, Silale A, Traynor D, Williams TD, Thomason PA, et al. Rapid and efficient genetic engineering of both wild type and axenic strains of *Dictyostelium discoideum*. *PLoS ONE* 2018; **13**.

4. Bates D, Mächler M, Bolker B, Walker S. Fitting Linear Mixed-Effects Models Using lme4. *J Stat Softw* 2015; **67**: 1–48.

5. Kuznetsova A, Brockhoff PB, Christensen RHB. lmerTest Package: Tests in Linear Mixed Effects Models. *J Stat Softw* 2017; **82**: 1–26.

6. R Core Team. R: A language and environment for statistical computing. 2014. R Foundation for Statistical Computing, Vienna, Austria.

7. Camacho C, Coulouris G, Avagyan V, Ma N, Papadopoulos J, Bealer K, et al. BLAST+: architecture and applications. *BMC Bioinformatics* 2009; **10**: 421.

8. Andrews S. FastQC: a quality control tool for high throughput sequence data. 2010.

9. Wick RR, Judd LM, Gorrie CL, Holt KE. Unicycler: Resolving bacterial genome assemblies from short and long sequencing reads. *PLOS Comput Biol* 2017; **13**: e1005595.

10. Gurevich A, Saveliev V, Vyahhi N, Tesler G. QUAST: quality assessment tool for genome assemblies. *Bioinformatics* 2013; **29**: 1072–1075.

11. Parks DH, Imelfort M, Skennerton CT, Hugenholtz P, Tyson GW. CheckM: assessing the quality of microbial genomes recovered from isolates, single cells, and metagenomes. *Genome Res* 2015; **25**: 1043–1055.

12. Arkin AP, Cottingham RW, Henry CS, Harris NL, Stevens RL, Maslov S, et al. KBase: The United States Department of Energy Systems Biology Knowledgebase. *Nat Biotechnol* 2018; **36**: 566–569.

13. Kikuchi Y, Fukatsu T. Live imaging of symbiosis: spatiotemporal infection dynamics of a GFP-labelled *Burkholderia* symbiont in the bean bug *Riptortus pedestris*. *Mol Ecol* 2014; **23**: 1445–1456.

14. Inglis RF, Biernaskie JM, Gardner A, Kümmerli R. Presence of a loner strain maintains cooperation and diversity in well-mixed bacterial communities. *Proc R Soc B Biol Sci* 2016; **283**: 20152682.

15. Brock DA, Haselkorn TS, Garcia JR, Bashir U, Douglas TE, Galloway J, et al. Diversity of free-living environmental bacteria and their interactions with a bactivorous amoeba. *Front Cell Infect Microbiol* 2018; **8**.

16. Brock DA, Douglas TE, Queller DC, Strassmann JE. Primitive agriculture in a social amoeba. *Nature* 2011; **469**: 393–396.

17. Perreau J, Patel DJ, Anderson H, Maeda GP, Elston KM, Barrick JE, et al. Vertical transmission at the pathogen-symbiont interface: *Serratia symbiotica* and aphids. *mBio* 2021; **12**: e00359-21.
